# Race–Ethnicity and Depressive Symptoms Among U.S. Older Adults in the COVID-19 Pandemic: Uncovering the Counterbalancing Mechanisms

**DOI:** 10.1093/geroni/igad003

**Published:** 2023-01-31

**Authors:** Miao Li, Ye Luo

**Affiliations:** Department of Sociology, Anthropology, and Criminal Justice, Clemson University, Clemson, South Carolina, USA; Department of Sociology, Anthropology, and Criminal Justice, Clemson University, Clemson, South Carolina, USA

**Keywords:** Competitive mediation, Depressive symptoms, Health and Retirement Study, Race, Resilience

## Abstract

**Background and Objectives:**

Drawing on the counterbalancing framework, this study examined the counteracting roles of coronavirus disease (COVID)-related stressors (i.e., infection threat, family activity disruption, economic impact) and psychological resilience in explaining racial–ethnic disparities in depressive symptoms during the COVID-19 pandemic.

**Research Design and Methods:**

A competitive mediation model was fitted using nationally representative data from the Health and Retirement Study COVID-19 Project, which were collected in June 2020 (*N* = 1,717). A competitive mediation model was specified within which the associations between race–ethnicity categories and depressive symptoms were mediated by infection threat, family activity disruption, economic impact, and psychological resilience. A list of pre-COVID covariates and pre-COVID depressive symptoms were adjusted for in this model.

**Results:**

Infection threat, family activity disruption, economic impact, and psychological resilience were all higher among non-Hispanic Blacks and Hispanics than among non-Hispanic Whites. Economic impact had a positive whereas psychological resilience had a negative direct effect on depressive symptoms net of pre-COVID covariates and pre-COVID depressive symptoms. Mediation analyses revealed that, compared to non-Hispanic Whites, non-Hispanic Blacks and Hispanics had higher depressive symptoms due to their higher family activity disruption and higher economic impact, but their higher levels of psychological resilience also reduced depressive symptoms. The counteracting indirect effects offset each other, resulting in a null total effect of race–ethnicity on depressive symptoms.

**Discussion and Implications:**

These findings suggest that interventions addressing the mental health impact of COVID should consider race/ethnicity-specific vulnerabilities and resilience. Future studies need to consider the complex and potentially counterbalancing mechanisms linking race–ethnicity and mental health.

**Translational Significance:** It is important to examine counteracting mechanisms underlying the paradoxical finding that racial–ethnic minorities had comparable mental health as Whites, even during the coronavirus disease (COVID) pandemic. This study found that, compared to Whites, minority older adults had higher depressive symptoms due to higher COVID-related risk exposure (family activity disruption and economic impact), but their higher levels of psychological resilience reduced depressive symptoms. Such findings suggest that interventions addressing social isolation and economic impact during the COVID-19 pandemic should particularly focus on minority vulnerability, whereas interventions boosting individual resilience might need to attend to White vulnerability.

## Background and Objectives

The global pandemic of coronoavirus disease (COVID)-19 has generated significant economic, psychological, and health burdens on the older population (Li & Mutchler, 2020). Moreover, racial–ethnic minority groups were disproportionately affected by COVID-19 in terms of exposure risk, hospitalization/death rates, economic hardship, and disrupted social relations (Gauthier et al., 2021; Taylor et al., 2022). The colored nature of COVID-19 triggered a growing literature evaluating racial–ethnic disparities in mental health during the COVID pandemic, which nevertheless generated inconsistent evidence. For instance, although non-Hispanic Black and Hispanic older adults showed greater concerns about the COVID-19 pandemic than Whites, they reported lower or similar levels of distress, and their psychological well-being was less sensitive to perceived COVID-19 threat (Bui et al., 2020; Hamler et al., 2022; Lin & Liu, 2021). Surprisingly, there is still no study examining racial–ethnic differences in depressive symptoms among older adults during the first wave of the COVID-19 pandemic in the United States.

The inconsistent findings on racial–ethnic disparities in mental health during the COVID-19 pandemic echoed study findings before the pandemic. For instance, the Black–White Paradox literature documented that, despite their disadvantaged social position, non-Hispanic Blacks tend to have similar or better mental health outcomes than Whites (Tobin et al., 2022). Although less prominent, similar paradoxical findings were reported on Hispanic-White mental health disparities (Breslau et al., 2005; Gallo et al., 2009; Walsemann et al., 2009). Moreover, a recent study suggested that non-Hispanic Blacks and Hispanics are less likely to be upset by exposure to stressors than Whites (Brown et al., 2018). Taken together, these studies suggest that counteracting mechanisms may exist in the stress process linking race–ethnicity and depressive symptoms. To better understand racial–ethnic differences in depressive symptoms during the COVID-19 pandemic, we need to disentangle the stress process by simultaneously considering the roles of stressor exposure and protective factors. The objective of the present study is to examine racial–ethnic differences in depressive symptoms among U.S. older adults during the first wave of the COVID-19 pandemic, with a focus on counterbalancing effects of COVID-related stressors and psychological resilience.

### Theory and Hypotheses

The guiding theoretical framework for our study is the counterbalancing model, which was developed by Louie and Wheaton (2019) in their application of stress process theory to explain the Black–White Paradox in mental health. A key tenet of the stress process theory is that social positions, including race–ethnicity, influence mental health by structuring exposure to stressors and access to coping resources (Pearlin et al., 2005). Earlier studies on racial disparities in mental health primarily focused on documenting the generally higher levels of stressor exposure among racial–ethnic minorities and the possible penalties these stressors may exert on their mental health. Another equally important element of the stress process model, the distribution of coping resources among groups, was either not considered or was assumed to follow a reversed pattern as stressor exposure (i.e., lower among minorities than among Whites). This is why findings about similar or better mental health outcomes among non-Hispanic Blacks compared to Whites were termed as a “paradox.” In explaining this paradoxical pattern, the counterbalancing model conceptualizes coping resource as an independent and additive process to the stressor exposure and explicitly considers the possibility that some advantages in coping resources among racial minorities may offset the deleterious effects of their disproportionate exposure to stressors (Louie & Wheaton, 2019). According to the counterbalancing model, coping recourses were related to but not completely determined by stressor exposure. Unique collective experiences and culture may help nourish resilience factors among the disadvantaged minorities. Studies noted that advantages in some coping resources, such as religiosity and self-esteem, counterbalanced the negative impacts of stressors on mental health for racial–ethnic minorities (Louie et al., 2022; Mouzon, 2017).

Although it was initially developed to explain the Black–White paradox in mental health, the counterbalancing model provides a general framework for understanding the complex stress processes that influence racial–ethnic disparities in mental health. Given that different racial–ethnic groups have different stress exposure and coping resources, it is necessary and important to apply this counterbalancing model to examine mental health disparities for other minority groups. Documenting the counterbalancing mechanisms will help us achieve a more nuanced and accurate estimation of racial–ethnic disparities, regardless of whether these mechanisms may produce a “paradox” or not. In this study, we focused on the counterbalancing effects of three COVID-related stressors (i.e., infection threat, economic impact, and family activity disruption) and an important coping resource during disruptive times—psychological resilience.

### COVID-Related Stressors

Infection threat, economic impact, and disrupted social activity are reoccurring themes in the literature and public narratives about the impact of COVID-19. Older adults are not immune to these stressors. Fear and anxiety over virus contraction become part of the daily life for many people as living with constant practice of precautions becomes a new norm (Arora et al., 2020). Economically, older adults faced risks of employment disruption, income loss, and food insecurity in the economic brunt of the pandemic (Garcia et al., 2021; Li & Mutchler, 2020). Moreover, restrictive measures such as social distancing and lockdowns inevitably disrupted people’s regular social activities, which may cause readjustment stress, feelings of relative deprivation, feelings of uncertainty and/or powerlessness, and social isolation (Adepoju et al., 2021; Henning-Smith, 2020; Ito et al., 2021). Infection threat, economic impact, and family activity disruption, therefore, represent three important COVID-related stressors that may simultaneously (and differentially) compromise the mental health of older adults during the pandemic.

Moreover, recent studies found that these COVID-related stressors are unequally distributed among racial–ethnic groups. For example, racial–ethnic minorities are more likely to have infected family members or experience the death of loved one due to COVID-19 (Dai et al., 2021; Gorges & Konetzka, 2021; Karaye & Horney, 2020; Webb Hooper et al., 2020; Zelner et al., 2021). Insofar as infection threat creates mental stress, as was found in existing studies, higher infection threat among racial–ethnic minorities means that levels of depressive symptoms among minority groups might be higher. Further, compared to non-Hispanic Whites, non-Hispanic Blacks and Hispanics were more likely to experience economic hardships during the pandemic, such as food insufficiency, job loss, and income loss (Nagata et al., 2021; Perry et al., 2021). Higher levels of COVID-triggered economic hardships among racial–ethnic minorities may thus put them at higher levels of depressive symptoms. Finally, it is also possible that non-Hispanic Blacks and Hispanics bear a disproportionate burden from disrupted family activities amid social distancing and lockdowns, given the prominence of kinship networks and generally higher frequency of face-to-face contact with relatives in the pre-pandemic life among these minority groups (Ajrouch et al., 2001; Katz et al., 2020). Racial–ethnic disparities in family activity disruption may translate into disparities in depressive symptoms, as family activity disruption poses threat to mental health by triggering readjustment stress, feelings of relative deprivation, feelings of uncertainty and powerlessness, and/or social isolation. As such, we hypothesize that: (1) *compared to non-Hispanic Whites, non-Hispanic Blacks and Hispanics have higher infection threat, family activity disruption, and economic impact*; (2) *depressive symptoms are positively associated with infection threat, family activity disruption, and economic*; (3) *non-Hispanic Blacks and Hispanics have higher depressive symptoms than non-Hispanic Whites because they experience higher infection threat, family activity disruption, and economic impact*.

### Psychological Resilience

A major contribution of the counterbalancing model is that it challenges the monolithic view of minority mental health deficit and highlights the protective effects of some unique coping resources of racial–ethnic minorities (Gallo et al., 2009; Louie et al., 2022). An important coping resource for racial–ethnic minorities is psychological resilience, an internal capacity for successful adaptation to adversity. Psychological resilience protects mental health through a variety of mechanisms, such as activating positive reappraisal, sustaining hope and optimism, meaning making, and facilitating support seeking (King et al., 2019; Wagnild & Collins, 2009).

Previous research suggested that minority groups tend to have more psychological strength when facing adversities and are more likely to successfully adapt than Whites (Assari et al., 2015; Louie & Wheaton, 2019; Ryff et al., 2003). Some argue that such psychosocial capacity for resilient coping among racial–ethnic minorities stems from their collective experience of sustained hardships and/or unique cultural capital. For instance, surviving the relentless history of slavery and subsequent 100-year legalized discrimination, the Black community has forged racial socialization practices that instill cultural pride, prepare for bias/discrimination, promote self-worth, emphasize trust in divine control, and build up a positive collective identity (Okeke-Adeyanju et al., 2014). These elements in the Black culture can contribute to a higher level of psychological resilience among Blacks, which may in turn protect their mental health.

Similarly, previous research also noted that certain Hispanic cultural orientations and practices such as *allocentrism*, *familism*, and *simpatia* may contribute to the Hispanic resilience (Gallo et al., 2009). *Allocentrism* is a cultural orientation that emphasizes focusing on other people rather than oneself, whereas *familism* is a cultural orientation that prioritizes family instead of individual. Both *allocentrism* and *familism* foster strong social bonding and promote collectivistic coping strategies such as social support, and therefore, they were associated with lower levels of perceived stress (Shen, 2016). *Simpatia* refers to the preference of warm and positive social interactions and avoidance of conflict and negativity (Acevedo et al., 2020). *Simpatia* facilitates positive social interactions and emotional regulation. Together, these cultural orientations and practices are conducive to the development of psychological resilience among the Hispanic community.

In sum, previous research on minority resilience suggests that minority groups may have a lower level of depressive symptoms than Whites due to their higher levels of psychological resilience. We, therefore, propose the following hypotheses: (4) *non-Hispanic Blacks and Hispanics have higher psychological resilience compared to non-Hispanic Whites*; (5) *depressive symptoms are negatively associated with psychological resilience*; and (6) *non-Hispanic Blacks and Hispanics have lower depressive symptoms due to their higher psychological resilience*.

### A Competitive Mediation Model

COVID-related stressors and psychological resilience represent opposing mechanisms linking race–ethnicity and depressive symptoms, with the former suggesting a minority deficit while the latter a minority asset. The counterbalancing effects from these mediators may offset each other, resulting in a null total effect. In such a case, the traditional strategy for mediation analyses, which seeks to first establish a mediation condition by identifying a significant total effect, will erroneously reject the theoretically important pathways that could otherwise be revealed. To address this issue, Zhao and colleagues proposed a competitive mediation model, which could be implemented in the structural equation modeling framework (Zhao et al., 2010). The competitive mediation approach replaced the traditional “Baron-Kenny + Sobel” approach with one-step test of the indirect effects. In other words, to establish mediation, “all that matters is that the indirect effect is significant” (Zhao et al., 2010, p. 204).

A competitive mediation, also known as inconsistent mediation, refers to the situation in which the indirect path and direct path (or multiple indirect paths) bear different signs in relating to the outcome. Competitive mediation indicates the existence of a “suppression effect,” a situation in which the (direct or indirect) association between an independent variable and a dependent variable becomes more visible after adjusting for a variable known as the “suppressor.” Often the suppressor represents a counteracting pathway that bears an opposite sign to the direct path or other indirect paths (MacKinnon et al., 2000). The opposing effect of the suppressor, being uncontrolled in the model, will be blended into the estimated effect of the independent variable and result in a reduced or null estimate. Consider the relationship between condom availability and the rate of sexually transmitted disease (STD). Theoretically, condom availability would lower the STD rate by promoting safer sex (Mediator 1). However, condom availability may also increase STD rate via lower perceived risk (Mediator 2) by creating a “get-out-of-jail-free card.” The two competing mediators, safer sex and lower perceived risk, are mutual suppressors. Without adjusting for perceived risk, the indirect (negative) effect of condom availability on STD via safer sex will not be significant. In contrast, without adjusting for safer sex, the indirect (positive) effect of condom availability on STD via lower perceived risk will not be significant either.

**Figure 1.**
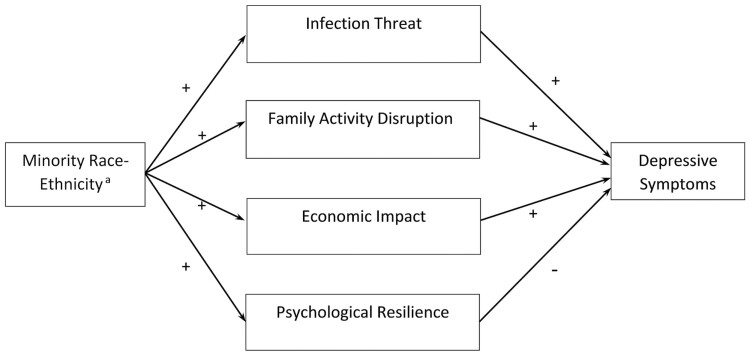
Conceptual framework for pathways linking race and depressive symptoms during the COVID-19 pandemic. ^a^Reference group: non-Hispanic White; focal minority groups: non-Hispanic Black and Hispanic.

By adjusting for the suppressor, we could disentangle competing mechanisms linking the independent variable and the dependent variable. Using this suppressor identification approach, Louie and Wheaton (2019) successfully disentangled the counterbalancing mediating effects of traumatic exposure and self-esteem in the relationship between race and mental health. Compared to the stepwise approach adopted by them, a competitive mediation model within the structural equation model framework provides a more straightforward, intuitive, and parsimonious approach that could estimate all parameters simultaneously. This study tested Hypotheses (1)–(6) in one single competitive mediation model that is illustrated in Figure 1.

## Research Design and Methods

### Data

Analyses of this study were based on the midterm release data of the Health and Retirement Study (HRS) COVID-19 Project. Started in 1992, the HRS is the largest and most representative ongoing biennial survey of U.S. adults aged 50 and older (Sonnega et al., 2014). As part of the HRS 2020, the HRS COVID-19 Project administered a COVID-19 module to an approximately 25% random subsample of the original HRS sample in June 2020. Telephone interviews were conducted due to the necessity for social distancing. By the time of this data release, the field work was still under way and the response rate was 62%. Among all respondents (*N* = 3,266) from this release, we restricted our analyses to a random subsample that completed the Leave-Behind Questionnaire of the year and had valid survey weights (*N* = 1,907). This is because some of the key variables (e.g., family activity disruption, etc.) were only included in the Leave-Behind Questionnaire. After the list-wise deletion of respondents with missing data on relevant variables, the final analytic sample size was 1,717. These respondents’ pre-pandemic HRS data were also used to measure some of the sociodemographic covariates, their pre-pandemic physical health, and depressive symptoms (see Table 1).

**Table 1.** Weighted Sample Characteristics, Health and Retirement Study 2020 COVID-19 Project (*N* = 1,717)

Characteristic	Race–ethnicity				*p* Value
	Non-Hispanic White (*n* = 1,146)	Non-Hispanic Black (*n* = 284)	Hispanic (*n* = 203)	Other (*n* = 84)	
Depressive symptoms (2020), mean ± *SD*	0.94 ± 1.40	1.38 ± 2.60	1.36 ± 2.56	0.97 ± 1.54	^**^
Depressive symptoms (2018), mean ± *SD*	1.01 ± 1.46	1.39 ± 2.72	1.46 ± 2.82	1.10 ± 1.57	^*^
Infection threat (2020), mean ± *SD*	0.51 ± 0.65	0.77 ± 1.24	0.65 ± 0.96	0.67 ± 0.72	^***^
Family activity disruption (2020), mean ± *SD*	1.71 ± 1.23	2.31 ± 2.40	2.13 ± 1.99	1.74 ± 1.46	^***^
Economic impact (2020), mean ± *SD*	0.37 ± 0.58	0.67 ± 1.74	1.03 ± 1.65	0.42 ± 0.69	^***^
Psychological resilience (2020), mean ± *SD*	4.08 ± .80	4.43 ± 1.31	4.31 ± 1.39	4.48 ± 0.76	^***^
Male, %	46.1	35.7	42.3	62.6	^**^
Education, mean ± *SD*	14.08 ± 2.08	13.30 ± 3.83	11.65 ± 5.15	14.37 ± 2.50	^***^
Age (2020), mean ± *SD*	68.56 ± 8.27	67.08 ± 12.06	66.15 ± 10.79	62.92 ± 7.16	^***^
Marital status (2020), %					
Married	65.7	38.6	63.1	70.7	^***^
Divorced/separated/widowed	29.7	45.2	32.4	19.4	
Never married	4.6	16.2	4.5	9.9	
Income (in thousands, 2016), mean ± *SD*	12.02 ± 16.93	7.80 ± 14.20	7.13 ± 15.48	9.69 ± 7.59	^***^
Self-rated health (2018), mean ± *SD*	2.59 ± .87	2.94 ± 1.46	3.11 ± 1.20	2.66 ± 1.05	^***^
Pre-existing conditions (2018), mean ± *SD*	2.65 ± 1.50	3.12 ± 2.47	2.55 ± 1.86	2.09 ± 1.76	^*^
Functional limitations (2018), mean ± *SD*	2.45 ± 2.33	3.55 ± 4.97	2.91 ± 3.90	2.18 ± 2.90	^***^
Worked for pay since the outbreak of COVID (2020), %	34.9	29.0	38.8	56.6	^**^
Compensatory virtual contact with family (2020), %	33.8	40.0	29.8	49.1	^*^

*Note*: *SD* = standard deviation.

^*^ *p* < .05.

^**^ *p* < .01.

^***^ *p* < .001.

### Measurements

Depressive symptoms were measured as the sum of affirmative answers to the eight-item short form of the Center for Epidemiological Studies—Depression scale (CES-D) which asked respondents whether they had the following experiences during the past week: everything was an effort; sleep was restless; felt happy (reverse coded); felt lonely; enjoyed life (reverse coded); felt sad; had a lot of energy (reverse coded); and could not get going.

#### Race and ethnicity

Based on the HRS survey questions on race and ethnicity, this study constructed four mutually exclusive categories: non-Hispanic Black, non-Hispanic White, Hispanic, and other races

#### Infection threat

Measurement for infection threat was constructed based on the following four survey questions: “Have you had or do you now have COVID-19?”; “Has anyone in your household other than you been diagnosed with COVID-19?”; “Has anyone else you know been diagnosed with COVID-19?”; and “Has anyone you know died from COVID-19?” Positive answers to these questions were summed to create a global score of infection threat (ranges from 0 to 4).

#### Family activity disruption

Respondents reported whether they have experienced any of the following changes in five activities during the COVID pandemic: unable to visit a family member in a care facility, nursing home, or group home; family celebrations canceled or restricted; unable to visit a close family member who was in hospital; unable to attend in-person funeral or religious services for a family member or friend who died; and unable to visit family after the birth of a new baby. A summary score of social activity disruption was constructed by counting all positive answers (ranges from 0 to 5).

#### Economic impact

Respondents reported if they have experienced the following economic/financial changes since the start of the pandemic: experienced income decline; missed any regular payments on rent or mortgage; missed any regular payments on credit cards or other debt; missed any other regular payments such as utilities or insurance; could not pay medical bills; didn’t have enough money to buy food; had trouble buying food even though had money; and other material hardship. Positive answers to these experiences were summed to create a score of economic impact (ranges from 0 to 7).

#### Psychological resilience

The HRS COVID Module adopted a novel six-item COVD-19 resilience scale initially developed in the Age Well Study (Mother Institute, 2021). The scale asked respondents to rate how much the following statements describe their experiences since the COVID Pandemic: “I tend to recover quickly after difficult times like this one,” “I have learnt some positive things from this situation about myself,” “I found greater meaning in work or my other activities and hobbies,” “I now feel more in touch with people in my local community,” “I found new ways to connect socially with other people,” and “I am now more appreciative of things that I had taken for granted before.” This scale taps into self-efficacy (Item 1), active coping (Item 5), and positive cognitive reappraisal (Items 2, 3, 4, and 6), which are key components of psychological resilience (Iacoviello & Charney, 2014; van der Meer et al., 2018; Windle et al., 2011). Responses to each item were recorded on a 6-point Likert scale, ranging from “strongly disagree” to “strongly agree.” The mean score of responses ranged from 1 to 6, with higher score representing higher psychological resilience. For this sample, the Cronbach’s alpha reliability was 0.82.

#### Covariates

Models for mediators (i.e., infection threat, family activity disruption, economic impact, and psychological resilience) adjusted for age (in years), sex (female, male), education (in years), marital status (married, divorced/separated/widowed, never married), household income (in thousand dollars), physical disability (count of functional limitations), number of diagnosed pre-existing chronic conditions (cancer, hypertension, stroke, heart problems, lung disease, diabetes, and obesity), self-rated health (ranging from 1 = excellent to 5 = poor), worked for pay since the COVID-19 pandemic (yes, no), and depressive symptoms in 2018 (i.e., the sum score of the same 8-item CES-D scale mentioned earlier). Model for the outcome (i.e., depressive symptoms in 2020) additionally adjusted for compensatory virtual contact with family members, which was measured as a dummy variable indicating an increased amount of contact with children, grandchildren, and other family members outside their home by phone, email, facetime, Facebook, Skype, Zoom, or social media since the start of the pandemic.

### Analytic Strategy

Weighted sample statistics stratified by race–ethnicity were computed in Stata 17 (StataCorp, 2021). Racial–ethnic differences in sample characteristics were tested using Chi-square tests for categorical variables and ANOVA tests for continuous variables. We then fit a competitive mediation model that was illustrated in Figure 1 using Mplus 8 (Muthén & Muthén, 1998–2017): a linear regression for depressive symptoms with race–ethnicity (White as the reference group) as the independent variable, adjusting for all mediators and covariates; whereas a linear regression for each of the mediators with race–ethnicity as the independent variable, adjusting for covariates. Given the skewed distribution of depressive symptoms with potential overdispersion, we further performed a robust test by specifying a negative binomial model for depressive symptoms. This alternative model specification did not yield substantively different findings. We, therefore, reported results from the competitive mediation model with a linear regression specification for depressive symptoms. Results from the model with negative binomial regression for the outcome were reported in Supplementary Tables 1 and 2.

To account for potential mediator-outcome confounding due to relationships between the mediators (VanderWeele, 2015), we specified correlated error terms among the mediators. Mediation effects were tested using the bias-corrected bootstrap confidence interval method with 10,000 replications (Williams & Mackinnon, 2008). An interaction term between family activity disruption and compensatory virtual family contact was added to the outcome model with the expectation that the impact of family activity disruption on depressive symptoms might be moderated by the compensatory virtual contacts, as was documented in other studies (Gabbiadini et al., 2020; Marinucci et al., 2022; Stuart et al., 2021).

## Results

### Descriptive Statistics

Table 1 shows the sample characteristics by racial–ethnic groups. Compared to non-Hispanic Whites, non-Hispanic Blacks and Hispanics scored higher on depressive symptoms in both 2020 and 2018, face higher infection threats, report more disruptions in family activities, and bear heavier burden of economic impact. Both non-Hispanic Blacks and Hispanics displayed higher level of psychological resilience than Whites. In terms of sociodemographic background, non-Hispanic Blacks and Hispanics were more likely to be unmarried, have lower levels of education and pre-pandemic income, and are less likely to work for pay during the COVID pandemic. Regarding pre-pandemic health status, non-Hispanic Blacks and Hispanics reported worse self-rated health and more functional limitations than non-Hispanic Whites. Pre-existing conditions were higher among non-Hispanic Blacks than among non-Hispanic Whites. Notably, depressive symptoms did not see much change across the 2018 and 2020 measures for all racial–ethnic groups. non-Hispanic Blacks were particularly more likely to practice compensatory virtual contact with family members than both non-Hispanic Whites and Hispanics.

### Competitive Mediation Analysis

Table 2 shows the path coefficients. After controlling for demographic covariates and depressive symptoms in 2018, non-Hispanic Blacks and Hispanics had higher levels of infection threat, family activity disruption, economic impact, and psychological resilience. Among the mediators, infection threat was not associated with depressive symptoms. Family activity disruption was significantly associated with higher levels of depressive symptoms for respondents who did not report increased compensatory virtue contact with family members *b* = 0.09, bootsrap 95% CI = [0.03, 0.15], as was indicated by the significant negative interaction term between the two variables *b* = –0.11, bootstrap 95% CI = [–0.21, –0.01]. Economic impact was significantly associated with higher levels of depressive symptoms *b* = 0.16, bootstrap 95% CI = [0.06, 0.26]. Psychological resilience was negatively associated with depressive symptoms *b* = –0.13, bootstrap 95% CI = [–0.20, –0.05]. Our robust test with a negative binomial model for depressive symptoms yielded very similar findings, except that the interaction term between family activity disruption and compensatory family virtual contact was no longer significant (see Supplementary Table 1).

**Table 2.** Medication Analysis for Racial Disparities in Depressive Symptoms During the COVID-19 Pandemic, Health and Retirement Study 2020 COVID-19 Project (*N* = 1,717)

Variable	Pathways^a^				Depressive symptoms 2020^b^
	Infection threats	Family activity disruption	Economic impact	Psychological resilience	
Non-Hispanic Black^c^	**0.41** ^***^	**0.72** ^***^	**0.27** ^***^	**0.45** ^***^	−0.16
	**[0.30, 0.52]**	**[0.52, 0.92]**	**[0.12, 0.42]**	**[0.33, 0.56]**	[−0.36, 0.04]
Hispanic^c^	**0.32** ^***^	**0.61** ^***^	**0.43** ^***^	**0.51** ^***^	0.13
	**[0.19, 0.45]**	**[0.37, 0.85]**	**[0.26, 0.60]**	**[0.34, 0.67]**	[−0.11, 0.37]
Other^c^	0.07	0.19	−0.08	**0.44** ^***^	0.03
	[−0.09, 0.23]	[−0.19, 0.56]	[−0.25, 0.08]	**[0.25, 0.63]**	[−0.30, 0.37]
Infection threats					0.02
					[−0.08, 0.11]
Family activity disruption					**0.09** ^**^
					**[0.03, 0.15]**
Economic impact					**0.16** ^***^
					**[0.06, 0.26]**
Psychological resilience					−0.13^***^
					**[**−**0.20,** −**0.05]**
Family activity disruption × Compensatory family virtual contact					−0.11^*^
					**[**−**0.21,** −**0.01]**

*Notes*: Bias-corrected bootstrap confidence intervals in brackets. Boldface indicates statistically significant estimates.

^a^ Models adjusted for age, sex, education, marital status, household income, physical disability, pre-existing chronic conditions, self-rated health, worked for pay since the outbreak of pandemic, and baseline depressive symptoms (2018).

^b^ Other than above covariates controlled in the pathway models, the outcome model additionally adjusted for compensatory family virtual contact and its interaction with family activity disruption.

^c^ Reference group: White.

^*^ *p* < .05.

^**^ *p* < .01.

^***^ *p* < .001.

Table 3 shows the total, direct, and indirect effects of race–ethnicity on depressive symptoms. First, the total effects show that neither non-Hispanic Blacks nor Hispanics had significantly higher depressive symptoms than Whites. However, the indirect effect estimates reveal a “competitive mediation” pattern, in which the indirect pathways through family activity disruption and economic impact were contrary to the indirect pathway via psychological resilience. Specifically, compared to non-Hispanic Whites, non-Hispanic Blacks tend to have higher depressive symptoms due to their higher family activity disruption *b =* 0.064, bootstrap 95% CI = [0.015, 0.112] and higher economic impact *b* =0.044, bootstrap 95% CI = [0.010, 0.079]. On the other hand, non-Hispanic Blacks’ higher level of psychological resilience reduced their depressive symptoms *b* = –0.057, bootstrap 95% CI = [–0.093, –0.021]. After accounting for all pathways, the direct effect of non-Hispanic Black race on depressive symptoms was not significant. Together, the positive indirect effects and the negative indirect effect canceled each out, resulting in no significant overall difference between non-Hispanic Blacks and Whites in depressive symptoms (i.e., null total effect).

**Table 3.** Total, Direct, and Indirect Effects From Race–Ethnicity to Depressive Symptoms, Health and Retirement Study 2020 COVID-19 Project (*N* = 1,717)

Race^a^	Effect decomposition	Beta	Bias-corrected bootstrap confidence intervals
Non-Hispanic Black	Total indirect effect	0.057	[**−**0.022, 0.136]
	Infection threats	0.006	[**−**0.032, 0.044]
	Family activity disruption	**0.064** ^**^	**[0.016, 0.111]**
	Economic impact	**0.044** ^**^	**[0.010, 0.079]**
	Psychological resilience	**−0.057** ^**^	**[−0.093, −0.020]**
	Direct effect	**−**0.157	[**−**0.355, 0.042]
	Total effect	**−**0.100	[**−**0.285, 0.086]
Hispanic	Total indirect effect	0.065	[**−**0.023, 0.154]
	Infection threats	0.005	[**−**0.026, 0.035]
	Family activity disruption	**0.054** ^*^	**[0.011, 0.098]**
	Economic impact	**0.071** ^**^	**[0.016, 0.126]**
	Psychological resilience	**−0.065** ^**^	**[−0.108, −0.021]**
	Direct effect	0.129	[**−**0.109, 0.367]
	Total effect	0.194	[**−**0.046, 0.435]

*Note*: Boldface indicates statistically significant estimates.

^a^Reference group: Non-Hispanic White.

^*^ *p* < .05.

^**^ *p* < .01.

^***^ *p* < .001.

A similar “competitive mediation” pattern was observed for Hispanics. Compared to Whites, Hispanics tend to have higher depressive symptoms due to their higher family activity disruption *b* = 0.054, bootstrap 95% CI = [0.010, 0.098] and higher economic impact *b* = 0.071, bootsrap 95% CI = [0.016, 0.126]. On the other hand, Hispanics’ higher psychological resilience reduced their depressive symptoms *b* = –0.065, bootstrap 95% CI = [–0.107, –0.022]. After accounting for all pathways, the direct effect of being Hispanic on depressive symptoms was not significant. Together, the positive indirect effects and the negative indirect effect canceled each out, resulting in no significant overall difference between Hispanics and Whites in depressive symptoms (i.e., null total effect).

## Discussion and Implications

In explaining the Black–White mental health paradox, Louie and Wheaton (2019) developed the counterbalancing model and provided initial evidence supporting this model. They also called for more studies to explore how various coping resources among disadvantaged populations may counterbalance the deleterious mental health impacts from their disproportionate stressor exposure. Our study responded to this call by applying the counterbalancing model to understand race–ethnicity disparities in depressive symptoms among older adults in the context of the COVID-19 pandemic. It further extended the counterbalancing model to understand the Hispanic-White differences in depressive symptoms.

This is the first nationally representative study that examined how three important COVID-related risk factors (infection threat, family activity disruption, and economic impact), along with psychological resilience, may function as counteracting pathways linking race–ethnicity and depressive symptoms among U.S. older adults. Consistent with previous studies, this study shows that both the infection and economic burdens of COVID-19 are disproportionately borne by racial–ethnic minorities. Compared to non-Hispanic Whites, non-Hispanic Blacks and Hispanics faced higher levels of infection threat and economic impact. The study also provided new evidence regarding the unequal burdens of family activity disruption across racial–ethnic groups: non-Hispanic Blacks and Hispanics experienced more family activity disruptions than Whites during the pandemic.

The null findings regarding the total effects of race–ethnicity on depressive symptoms in the mediational analyses were in line with the counterbalancing model, that is, disadvantaged minority groups somehow had similar levels of mental health as Whites (Barnes & Bates, 2017; Louie & Wheaton, 2019). Results on indirect effects supported our hypotheses derived from the counterbalancing model: the COVID-related risk factors (i.e., family activity disruption and economic impact) and psychological resilience mediated the effects of minority race–ethnicity statuses on depressive symptoms in opposite directions, with negative effect of resilience offsetting the positive effects of family activity disruption and economic impact. Our finding of the counterbalancing mechanisms linking race and depressive symptoms among the older population during the COVID pandemic echoed the similar pattern among U.S. adolescents in the pre-pandemic period (Louie & Wheaton, 2019).

The findings of this study have both practical and theoretical implications. Practically, they suggest that policies and interventions alleviating the mental health impacts of the COVID pandemic should consider race-specific vulnerabilities. Racial–ethnic minorities are more vulnerable to socioeconomic impacts while Whites tend to have lower psychological resilience in coping with stressful situation. Interventions addressing social isolation and economic impact should particularly focus on minority vulnerability, while interventions boosting individual resilience might need to pay particular attention to White people. Theoretically, our findings about competitive mediation yielded fresh insights into the ongoing scholarly efforts to understand the paradoxical finding of similar or better mental health among socially disadvantaged racial–ethnic groups. Previous research that attempted to explain such paradoxes mostly focused on uncovering some “hidden” protective mechanisms among minorities (Louie et al., 2022; Mouzon, 2014, 2017). Findings from our study suggest that a global null association between race–ethnicity and mental health does not imply the absence of minority disadvantages and that searching for protective mechanisms should not prevent us from uncovering minorities’ disadvantages.

Although race–ethnicity was specified as an exogenous variable in the mediation model, we are not treating race–ethnicity as a cause but are following a widely practiced statistical approach to identify social factors that may explain racial–ethnic disparities in health. The mediation model provides us with a tool to demonstrate how racial–ethnic disparities in COVID-related stressors and psychological resilience, both of which are deeply rooted in social structure, explain disparities in depressive symptoms during the COVID-19 pandemic.

A few limitations should be considered in interpreting the findings from this study. First, the study period spanned only a few months into the outbreak of COVID-19. Given the unabated pandemic and growing racial disparities in infection and economic burdens, it remains to be seen how the accumulated burdens of infection threat and economic impact may in the long run contribute to racial disparities in mental health. Second, other than infection threat and economic impact, there might be other factors through which COVID-19 differentially impacts the mental health of racial–ethnic minorities, such as restricted social contact, neighborhood-level medical resource deprivation, and racism. However, we were unable to fully evaluate these potential mechanisms due to data limitations. Finally, given the limited sample size, we were unable to examine the mental health burden for people of other racial backgrounds, especially the Asian American subgroup, who were facing disproportionate levels of stress associated with discrimination and coronavirus-related stigma.

Despite these limitations, this study represents the first step toward understanding the unfolding racial–ethnic disparities in mental health in the context of COVID pandemic. Our findings call for future studies to continually monitor how the health, social, and economic ramifications of the global pandemic would shape the racial–ethnic disparities in mental health.

## Supplementary Material

igad003_suppl_Supplementary_Material

## References

[CIT0001] Acevedo, A. M., Herrera, C., Shenhav, S., Yim, I. S., & Campos, B. (2020). Measurement of a Latino cultural value: The Simpatía scale. Cultural Diversity and Ethnic Minority Psychology, 26, 419–425. doi:10.1037/cdp0000324 32105107

[CIT0002] Adepoju, O. E., Chae, M., Woodard, L., Smith, K. L., Herrera, L., Han, D., Howard, D. L., Dobbins, J., & Ory, M. (2021). Correlates of social isolation among community-dwelling older adults during the COVID-19 pandemic. Frontiers in Public Health, 9, 702965. doi:10.3389/fpubh.2021.702965 34956998 PMC8702646

[CIT0003] Ajrouch, K. J., Antonucci, T. C., & Janevic, M. R. (2001). Social networks among Blacks and Whites: The interaction between race and age. The Journals of Gerontology, Series B: Psychological Sciences and Social Sciences, 56(2), S112–S118. doi:10.1093/geronb/56.2.s112 11245365

[CIT0004] Arora, A., Jha, A. K., Alat, P., & Das, S. S. (2020). Understanding coronaphobia. Asian Journal of Psychiatry, 54, 102384–102384. doi:10.1016/j.ajp.2020.102384 33271693 PMC7474809

[CIT0005] Assari, S., Burgard, S., & Zivin, K. (2015). Long-term reciprocal associations between depressive symptoms and number of chronic medical conditions: Longitudinal support for Black–White health paradox. Journal of Racial and Ethnic Health Disparities, 2(4), 589–597. doi:10.1007/s40615-015-0116-9 26863563

[CIT0006] Barnes, D. M., & Bates, L. M. (2017). Do racial patterns in psychological distress shed light on the Black–White depression paradox? A systematic review. Social Psychiatry and Psychiatric Epidemiology, 52(8), 913–928. doi:10.1007/s00127-017-1394-9 28555381

[CIT0007] Breslau, J., Aguilar-Gaxiola, S., Kendler, K. S., Su, M., Williams, D., & Kessler, R. C. (2005). Specifying race-ethnic differences in risk for psychiatric disorder in a USA national sample. Psychological Medicine, 36(1), 57–68. doi:10.1017/s0033291705006161 16202191 PMC1924605

[CIT0008] Brown, L. L., Mitchell, U. A., & Ailshire, J. A. (2018). Disentangling the stress process: Race/ethnic differences in the exposure and appraisal of chronic stressors among older adults. The Journals of Gerontology, Series B: Psychological Sciences and Social Sciences, 75(3), 650–660. doi:10.1093/geronb/gby072 PMC7328036 29878196

[CIT0009] Bui, C. N., Peng, C., Mutchler, J. E., & Burr, J. A. (2020). Race and ethnic group disparities in emotional distress among older adults during the COVID-19 pandemic. Gerontologist, 61(2), 262–272. doi:10.1093/geront/gnaa217 PMC7799087 33367785

[CIT0010] Dai, C. L., Kornilov, S. A., Roper, R. T., Cohen-Cline, H., Jade, K., Smith, B., Heath, J. R., Diaz, G., Goldman, J. D., Magis, A. T., & Hadlock, J. J. (2021). Characteristics and factors associated with Covid-19 infection, hospitalization, and mortality across race and ethnicity. Clinical Infectious Diseases, 73(12), 2193–2204. doi:10.1093/cid/ciab154 33608710 PMC7929051

[CIT0011] Gabbiadini, A., Baldissarri, C., Durante, F., Valtorta, R. R., de Rosa, M., & Gallucci, M. (2020). Together apart: The mitigating role of digital communication technologies on negative affect during the COVID-19 outbreak in Italy [Brief Research Report]. Frontiers in Psychology, 11, 554678. doi:10.3389/fpsyg.2020.554678 33192807 PMC7609360

[CIT0012] Gallo, L. C., Penedo, F. J., Espinosa de los Monteros, K., & Arguelles, W. (2009). Resiliency in the face of disadvantage: Do Hispanic cultural characteristics protect health outcomes? Journal of Personality, 77(6), 1707–1746. doi:10.1111/j.1467-6494.2009.00598.x 19796063

[CIT0013] Garcia, M. A., Thierry, A. D., & Pendergrast, C. B. (2021). The devastating economic impact of COVID-19 on older Black and Latinx adults: Implications for health and well-being. The Journals of Gerontology, Series B: Psychological Sciences and Social Sciences, 77(8), 1501–1507. doi:10.1093/geronb/gbab218 PMC8690256 34850887

[CIT0014] Gauthier, G. R., Smith, J. A., García, C., Garcia, M. A., & Thomas, P. A. (2021). Exacerbating inequalities: Social networks, racial/ethnic disparities, and the COVID-19 pandemic in the United States. The Journals of Gerontology, Series B: Psychological Sciences and Social Sciences, 76(3), e88–e92. doi:10.1093/geronb/gbaa117 32756978 PMC7454830

[CIT0015] Gorges, R. J., & Konetzka, R. T. (2021). Factors associated with racial differences in deaths among nursing home residents with COVID-19 infection in the US. JAMA Network Open, 4(2), e2037431. doi:10.1001/jamanetworkopen.2020.37431 33566110 PMC7876590

[CIT0016] Hamler, T. C., Nguyen, A. W., Mouzon, D. M., Taylor, H. O., Qin, W., & Cobb, R. J. (2022). COVID-19 and psychological distress: Racial differences among middle-aged and older adults. Gerontologist, 62(5), 780–791. doi:10.1093/geront/gnac043 35349690 PMC9154222

[CIT0017] Henning-Smith, C. (2020). The unique impact of COVID-19 on older adults in rural areas. Journal of Aging & Social Policy, 32(4–5), 396–402. doi:10.1080/08959420.2020.1770036 32475255

[CIT0018] Iacoviello, B. M., & Charney, D. S. (2014). Psychosocial facets of resilience: Implications for preventing posttrauma psychopathology, treating trauma survivors, and enhancing community resilience. European Journal of Psychotraumatology, 5, 1. doi:10.3402/ejpt.v3405.23970 PMC4185137 25317258

[CIT0019] Ito, T., Hirata-Mogi, S., Watanabe, T., Sugiyama, T., Jin, X., Kobayashi, S., & Tamiya, N. (2021). Change of use in community services among disabled older adults during COVID-19 in Japan. International Journal of Environmental Research and Public Health, 18(3), 1148. doi:10.3390/ijerph18031148. https://www.mdpi.com/1660-4601/18/3/1148 33525441 PMC7908432

[CIT0020] Karaye, I. M., & Horney, J. A. (2020). The impact of social vulnerability on COVID-19 in the U.S.: An analysis of spatially varying relationships. American Journal of Preventive Medicine, 59(3), 317–325. doi:10.1016/j.amepre.2020.06.006 32703701 PMC7318979

[CIT0021] Katz, B., Turney, I., Lee, J. H., Amini, R., Ajrouch, K. J., & Antonucci, T. C. (2020). Race/ethnic differences in social resources as cognitive risk and protective factors. Research in Human Development, 17(1), 57–77. doi:10.1080/15427609.2020.1743809 34093091 PMC8174783

[CIT0022] King, B. M., Carr, D. C., & Taylor, M. G. (2019). Depressive symptoms and the buffering effect of resilience on widowhood by gender. Gerontologist, 59(6), 1122–1130. doi:10.1093/geront/gny115 30247641

[CIT0023] Li, Y., & Mutchler, J. E. (2020). Older adults and the economic impact of the COVID-19 pandemic. Journal of Aging & Social Policy, 32(4–5), 477–487. doi:10.1080/08959420.2020.1773191 32543304

[CIT0024] Lin, Z., & Liu, H. (2021). A national study of racial–ethnic differences in COVID-19 concerns among older Americans: Evidence from the Health and Retirement Study. The Journals of Gerontology, Series B: Psychological Sciences and Social Sciences, 77(7), e134–e141. doi:10.1093/geronb/gbab171 PMC9255937 34549286

[CIT0025] Louie, P., Upenieks, L., Erving, C. L., & Thomas Tobin, C. S. (2022). Do racial differences in coping resources explain the Black–White paradox in mental health? A test of multiple mechanisms. Journal of Health and Social Behavior, 63(1), 55–70. doi:10.1177/00221465211041031 34549645 PMC10624509

[CIT0026] Louie, P., & Wheaton, B. (2019). The Black-White paradox revisited: Understanding the role of counterbalancing mechanisms during adolescence. Journal of Health and Social Behavior, 60(2), 169–187. doi:10.1177/0022146519845069 31072135

[CIT0027] MacKinnon, D. P., Krull, J. L., & Lockwood, C. M. (2000). Equivalence of the mediation, confounding and suppression effect. Prevention Science, 1(4), 173–181. doi:10.1023/a:1026595011371 11523746 PMC2819361

[CIT0028] Marinucci, M., Pancani, L., Aureli, N., & Riva, P. (2022). Online social connections as surrogates of face-to-face interactions: A longitudinal study under Covid-19 isolation. Computers in Human Behavior, 128, 107102. doi:10.1016/j.chb.2021.107102

[CIT0029] Mother Institute. (2021). *The Age Well Study: Stress & resilience among residents of life plan communities during the pandemic—Year 4 report*. Retrieved 27 January 2022 from https://information.matherinstitute.com/age-well-study-year-4

[CIT0030] Mouzon, D. M. (2014). Relationships of choice: Can friendships or fictive kinships explain the race paradox in mental health? Social Science Research, 44, 32–43. doi:10.1016/j.ssresearch.2013.10.007 24468432

[CIT0031] Mouzon, D. M. (2017). Religious involvement and the Black–White paradox in mental health. Race and Social Problems, 9(1), 63–78. doi:10.1007/s12552-017-9198-9

[CIT0032] Muthén, L. K., & Muthén, B. O. (1998–2017). Mplus user’s guide. 8th ed. Muthén & Muthén.

[CIT0033] Nagata, J. M., Ganson, K. T., Whittle, H. J., Chu, J., Harris, O. O., Tsai, A. C., & Weiser, S. D. (2021). Food insufficiency and mental health in the U.S. during the COVID-19 pandemic. American Journal of Preventive Medicine, 60(4), 453–461. doi:10.1016/j.amepre.2020.12.004 33602534 PMC9067067

[CIT0034] Okeke-Adeyanju, N., Taylor, L. C., Craig, A. B., Smith, R. E., Thomas, A., Boyle, A. E., & DeRosier, M. E. (2014). Celebrating the strengths of black youth: Increasing self-esteem and implications for prevention. Journal of Primary Prevention, 35(5), 357–369. doi:10.1007/s10935-014-0356-1 25053261 PMC4152398

[CIT0035] Pearlin, L. I., Schieman, S., Fazio, E. M., & Meersman, S. C. (2005). Stress, health, and the life course: Some conceptual perspectives. Journal of Health and Social Behavior, 46(2), 205–219. doi:10.1177/002214650504600206 16028458

[CIT0036] Perry, B. L., Aronson, B., & Pescosolido, B. A. (2021). Pandemic precarity: COVID-19 is exposing and exacerbating inequalities in the American heartland. Proceedings of the National Academy of Sciences of the United States of America, 118(8), e2020685118. doi:10.1073/pnas.2020685118 33547252 PMC7923675

[CIT0037] Ryff, C. D., Corey, L. M. K., & Hughes, D. L. (2003). status inequalities, perceived discrimination, and eudaimonic well-being: Do the challenges of minority life hone purpose and growth? Journal of Health and Social Behavior, 44(3), 275–291. doi:10.2307/1519779 14582308

[CIT0038] Shen, J. J. (2016). Collectivistic coping, allocentrism, and stress [M.A., California State University, Long Beach]. ProQuest Dissertations & Theses Global. http://libproxy.clemson.edu/login?url=https://www.proquest.com/dissertations-theses/collectivistic-coping-allocentrism-stress/docview/1877615251/se-2

[CIT0039] Sonnega, A., Faul, J. D., Ofstedal, M. B., Langa, K. M., Phillips, J. W., & Weir, D. R. (2014). Cohort profile: The Health and Retirement Study (HRS). International Journal of Epidemiology, 43(2), 576–585. doi:10.1093/ije/dyu067 24671021 PMC3997380

[CIT0040] StataCorp. (2021). Stata Statistical Software: Release 17. StataCorp LLC.

[CIT0041] Stuart, J., O’Donnell, K., O’Donnell, A., Scott, R., & Barber, B. (2021). Online social connection as a buffer of health anxiety and isolation during COVID-19. Cyberpsychology, Behavior, and Social Networking, 24(8), 521–525. doi:10.1089/cyber.2020.0645 33601944

[CIT0042] Taylor, M. G., Carr, D. C., & Jason, K. (2022). Financial hardship and psychological resilience during COVID-19: Differences by race/ethnicity. The Journals of Gerontology, Series B: Psychological Sciences and Social Sciences, 77(7), E117–E122. doi:10.1093/geronb/gbab173 34604902 PMC9653000

[CIT0043] Tobin, C. S. T., Erving, C. L., Hargrove, T. W., & Satcher, L. A. (2022). Is the Black-White mental health paradox consistent across age, gender, and psychiatric disorders? Aging and Mental Health, 26(1), 196–204. doi:10.1080/13607863.2020.1855627 33291956 PMC8187467

[CIT0044] van der Meer, C. A. I., te Brake, H., van der Aa, N., Dashtgard, P., Bakker, A., & Olff, M. (2018). Assessing psychological resilience: Development and psychometric properties of the English and Dutch Version of the Resilience Evaluation Scale (RES). Frontiers in Psychiatry, 9, 169. doi:10.3389/fpsyt.2018.00169 PMC5968386 29867601

[CIT0045] VanderWeele, T. (2015). Explanation in causal inference: Methods for mediation and interaction. Oxford University Press.

[CIT0046] Wagnild, G. M., & Collins, J. A. (2009). Assessing resilience. Journal of Psychosocial Nursing and Mental Health Services, 47(12), 28–33. doi:10.3928/02793695-20091103-01 20000280

[CIT0047] Walsemann, K. M., Gee, G. C., & Geronimus, A. T. (2009). Ethnic differences in trajectories of depressive symptoms: Disadvantage in family background, high school experiences, and adult characteristics. Journal of Health and Social Behavior, 50(1), 82–98. doi:10.1177/002214650905000106 19413136 PMC2761954

[CIT0048] Webb Hooper, M., Nápoles, A. M., & Pérez-Stable, E. J. (2020). COVID-19 and racial/ethnic disparities. *Journal of the American Medical Association*, 323(24), 2466–2467. doi:10.1001/jama.2020.8598 PMC9310097 32391864

[CIT0049] Williams, J., & Mackinnon, D. P. (2008). Resampling and distribution of the product methods for testing indirect effects in complex models. Structural Equation Modeling, 15(1), 23–51. doi:10.1080/10705510701758166 20179778 PMC2825896

[CIT0050] Windle, G., Bennett, K. M., & Noyes, J. (2011). A methodological review of resilience measurement scales. Health and Quality of Life Outcomes, 9(1), 8. doi:10.1186/1477-7525-9-8 21294858 PMC3042897

[CIT0051] Zelner, J., Trangucci, R., Naraharisetti, R., Cao, A., Malosh, R., Broen, K., Masters, N., & Delamater, P. (2021). Racial disparities in coronavirus disease 2019 (COVID-19) mortality are driven by unequal infection risks. Clinical Infectious Diseases, 72(5), e88–e95. doi:10.1093/cid/ciaa1723 33221832 PMC7717213

[CIT0052] Zhao, X., Lynch, J., Xa, G., & Chen, Q. (2010). Reconsidering Baron and Kenny: Myths and truths about mediation analysis. Journal of Consumer Research, 37(2), 197–206. doi:10.1086/651257

