# Supplementary material for: Race–Ethnicity and Depressive Symptoms Among U.S. Older Adults in the COVID-19 Pandemic: Uncovering the Counterbalancing Mechanisms
Source: Innov Aging. 2023 Jan 31;7(2):igad003. doi: 10.1093/geroni/igad003 (PMC10024480; doi:10.1093/geroni/igad003)
Supplement: igad003_suppl_Supplementary_Material [file igad003_suppl_supplementary_material.docx]

*Innovation in Aging* Online Supplementary Material: Miao Li, & Ye Luo. Race-Ethnicity and Depressive Symptoms Among U.S. Older Adults in the COVID-19 Pandemic: Uncovering the Counterbalancing Mechanisms.

Supplementary Table 1. Robust Analysis with Negative Binomial Regression for Depressive Symptoms.

| **Characteristic** | **Pathways ^a^** | | | | **Depressive Symptoms 2020 ^b^** |
| --- | --- | --- | --- | --- | --- |
|  | **Infection Threats** | **Family Activity Disruption** | **Economic Impact** | **Psychological Resilience** |  |
| Black ^c^ | **0.41***** | **0.72***** | **0.27***** | **0.45***** | -0.13 |
|  | **[0.30, 0.52]** | **[0.52, 0.92]** | **[0.12, 0.42]** | **[0.33, 0.56]** | [-0.36, 0.04] |
| Hispanic ^c^ | **0.32***** | **0.61***** | **0.43***** | **0.51***** | 0.12 |
|  | **[0.19, 0.45]** | **[0.37, 0.85]** | **[0.26, 0.60]** | **[0.34, 0.67]** | [-0.11, 0.37] |
| Other ^c^ | 0.07 | 0.19 | -0.08 | **0.44***** | 0.00 |
|  | [-0.09, 0.23] | [-0.19, 0.56] | [-0.25, 0.08] | **[0.25, 0.63]** | [-0.30, 0.37] |
| Infection Threats | |  |  |  | 0.03 |
|  |  |  |  |  | [-0.06, 0.12] |
| Family Activity Disruption | |  |  |  | **0.08**** |
|  |  |  |  |  | **[0.03, 0.12]** |
| Economic Impact |  |  |  |  | **0.09***** |
|  |  |  |  |  | **[0.02, 0.15]** |
| Psychological Resilience |  |  |  |  | **-0.13***** |
|  |  |  |  |  | **[-0.20, -0.07]** |
| Family Activity Disruption × Compensatory Family Virtual Contact |  |  |  |  | -0.05 |
|  |  |  |  |  | [-0.14, 0.04] |
| Notes. Bias-corrected bootstrap confidence intervals in brackets. Boldface indicates statistically significant estimates; * p < 0.05, ** p < .01, *** p < .001  ^a^ Models adjusted for age, sex, education, marital status, household income, physical disability, pre-existing chronic conditions, self-rated health, worked for pay since the outbreak of pandemic, and baseline depressive symptoms (2018).  ^b^ Other than above covariates controlled in the pathway models, the outcome model additionally adjusted for compensatory family virtual contact and its interaction with family activity disruption.  ^c^ Reference group: White. | | | | | |

Supplementary Table 2. Robust Analysis for Total, Direct, and Indirect Effects with Negative Binomial Regression for Depressive Symptoms.

| **Race ^a^** | **Effect Decomposition** | **Beta** | **Bias-corrected Bootstrap Confidence Intervals** |
| --- | --- | --- | --- |
| Black | Total Indirect Effect | 0.029 | [-0.036, 0.094] |
|  | *Infection Threats* | 0.011 | [-0.026, 0.048] |
|  | *Family Activity Disruption* | **0.054**** | **[0.015, 0.092]** |
|  | *Economic Impact* | **0.023**** | **[0.001, 0.045]** |
|  | *Psychological Resilience* | **-0.058**** | **[-0.092, -0.025]** |
|  | Direct Effect | -0.132 | [-0.317, 0.052] |
|  | Total Effect | -0.103 | [ -0.277, 0.071] |
| Hispanic | Total Indirect Effect | 0.025 | [-0.044, 0.094] |
|  | *Infection Threats* | 0.009 | [-0.021, 0.038] |
|  | *Family Activity Disruption* | **0.046*** | **[0.011, 0.080]** |
|  | *Economic Impact* | **0.037**** | **[0.003, 0.071]** |
|  | *Psychological Resilience* | **-0.066**** | **[-0.107, -0.026]** |
|  | Direct Effect | 0.122 | [-0.085, 0.330] |
|  | Total Effect | 0.147 | [-0.056, 0.350] |
| Notes. Boldface indicates statistically significant estimates; * p < 0.05, ** p < .01, *** p < .001 | | | |
